# The attrition rate of licensed chiropractors in California: an exploratory ecological investigation of time-trend data

**DOI:** 10.1186/1746-1340-18-24

**Published:** 2010-08-12

**Authors:** Stephen M Foreman, Michael J Stahl

**Affiliations:** 1 Private practice of chiropractic, West Hills, California, USA

## Abstract

**Background:**

The authors hypothesized the attrition rate of licensed chiropractors in California has gradually increased over the past several decades. "Attrition" as determined for this study is defined as a loss of legal authority to practice chiropractic for any reason during the first 10 years after the license was issued. The percentage of license attrition after 10 years was determined for each group of graduates licensed in California each year between 1970 and 1998. The cost of tuition, the increase in the supply of licensed chiropractors and the ratio of licensed chiropractors to California residents were examined as possible influences on the rate of license attrition.

**Methods:**

The attrition rate was determined by a retrospective analysis of license status data obtained from the California Department of Consumer Affairs. Other variables were determined from US Bureau of Census data, survey data from the American Chiropractic Association and catalogs from a US chiropractic college.

**Results:**

The 10-year attrition rate rose from 10% for those graduates licensed in 1970 to a peak of 27.8% in 1991. The 10-year attrition rate has since remained between 20-25% for the doctors licensed between 1992-1998.

**Conclusions:**

Available evidence supports the hypothesis that the attrition rate for licensed chiropractors in the first 10 years of practice has risen in the past several decades.

## Background

A chiropractic license, issued by a governmental regulatory body, is the certification required to transition the graduate from academic study into the world of unsupervised practical application. The need for the license is paramount for without it the graduate remains a highly educated layman without unrestricted clinical practice rights. The ongoing maintenance of an active license in California requires several duties; the payment of an annual fee, the time and expense of yearly continuing education study and practicing within the guidelines of the license [1]. Failure to meet anyone of these duties can result in license restriction, suspension or revocation.

The loss of legal practice rights for any reason represents a major interruption in the chiropractor's professional career and may signify their complete exit from the profession. Reasons for license loss vary greatly and can include a move to another license jurisdiction, personal illness or disability, employment in other professions/jobs, disciplinary action against the license for improper behavior, retirement and even death of the doctor. The authors expected these various non-financial reasons for license loss to remain fairly consistent over the years.

The authors hypothesized the current attrition rate of licensed chiropractors has not been consistent and is now higher than the rate observed in past years. A rising attrition rate of licensees may be linked to a number of forces such as changes in population, an oversupply of chiropractors, changes in reimbursement, the cost of education and general dissatisfaction with the profession. Detection and documentation of a rising attrition rate within the profession may stimulate investigation to identify the causes and help set educational and license policies.

Our examination of the license attrition rates required the selection of a point in time to represent an earlier than expected loss of practice rights. The ten-year anniversary of initial license issuance was chosen for each yearly license group as this appeared to allow time for the newly licensed doctor to work with an experienced doctor, gain professional experience and business skills, obtain funding to open an office and, if desired, establish his/her own practice. The passage of 10 years also allowed time for the doctor to increase the number of patients they serve and become financially established in the community. A loss of practice rights in less than 10 years was believed to possibly represent a premature departure from the profession and inconsistent with the time, effort and expense devoted to education and earning a license.

## Methods

The authors hypothesized the trend of license attrition was rising during the study period from 1970 to 1998 and chose to observe the actual time-trend with an ecologically based exploratory analysis [2]. Collection of the data-points for the time-trend analysis required a review of the current license status for each chiropractic license issued between 1970 and 1998. The California Department of Consumer Affairs (DCA) maintains an online searchable database [3] that contains the current license status of all licensed chiropractors in California. California was chosen for study for a number of reasons including the large number of chiropractic colleges in the State during the past 28 years (n = 8), the large State population, its geographic size and the fact California has the greatest number of licensed chiropractors out of the estimated 69,000 chiropractors in the United States [4].

A "valid" status license allows the chiropractor to legally provide care. Other DCA license categories that do not allow licensed practice include "revoked, cancelled, inactive, voluntary suspension, forfeiture and deceased." The doctor may choose to reactivate a license that is inactive or in forfeiture status. The data obtained for this study from DCA does not allow determination of issues such as which doctors may have allowed their license to expire and are now practicing in other states, which doctors are in full-time versus part-time practice or the identity of doctors that may have left practice but are now employed in academia or related areas.

**Figure 1:**
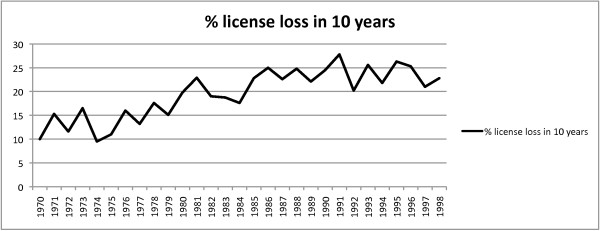
**Percentage of license attrition, 1970-1998**. A graphical depiction of the percentage of chiropractors without practice rights 10 years after the license was issued. The range was 10% in 1970 and a peak of 27.8% in 1991. The 10-year attrition rate has since remained between 20-25% for the doctors licensed between 1992-1998.

The authors obtained data from the DCA database for each chiropractor licensed between January 1, 1970 and November 7, 1998 (n = 14,922). The doctors licensed during 1970 (n = 126) were grouped and sorted according to the date their license expired and was listed as other than "valid." Analysis of the group licensed during 1970 revealed 12 doctors listed as other than "valid" for an attrition rate of 10% between 1/1/70 and 12/31/79. This grouping and sorting process was repeated for those graduates licensed each year until 1998. The 10-year attrition percentage was calculated for each yearly license group and the results were plotted (Figure 1). Analysis of the DCA database revealed a higher attrition rate for those doctors licensed in the 1980s and 1990s compared to their counterparts licensed in the 1970s. Attrition after 10 years for those licensed during 1970 was 10% and this rate continued to trend upward to a peak of 27.8% by 1991, an increase of 178%. The 10-year attrition rate since 1991 has reduced from its peak but remained between 20-25% for the doctors licensed between 1992-1998.

## Analysis

### Potential influences on the attrition rate trend

Documenting factors that may have contributed to increasing attrition rate may help identify root causes and shape future public and professional policy decisions. Although the ultimate causes of the increased attrition for each individual are unknown, previously published studies of the chiropractic profession provided insight and reasonable areas for future investigation. For example, Mior and Laporte's [5] economic and resource assessment of chiropractors practicing in Ontario, Canada between 1990 and 2004 revealed a "long-run oversupply of doctors that outpaced the increase in population." The number of registered chiropractors in Ontario doubled during their study period. The oversupply situation was paralleled by a reduction of annual net income from $97,892 in 1992-93 to $48,900 in 2002-03. Mior and Laporte concluded, "Chiropractors who cannot meet their operating costs and achieve a reasonable return on their investment in training will drop out of the market." These findings led us to investigate changes in the ratio of the domestic population and the number of licensed chiropractors in California during our study period.

Information from the United States Bureau of Census provided the percentage of change in California population each year between 1970 and 1998 [6]. California experienced a growth in residential population during each year between 1970 and 1998, the largest being 2.6% in 1981 and the lowest being 0.7% in 1995. The total residential population between 1970 and 1998 rose 65.1% from 19,971,071 to 32,987,675. The steady and gradual population increase each year would argue against it as a catalyst in the rising attrition rate for chiropractors. The steady change in residential population led us to investigate the changes in the number of licensed chiropractors in California since 1970.

### Changes in the number of licensed chiropractors

Mior and Laporte [5] noted a doubling of the licensed chiropractors in their Canadian study and a resultant reduction in both doctor/patient ratio and net annual income. These findings directed our study in California. The DCA database was reviewed to determine the total number of valid chiropractic licenses in California each year between 1970 and 1998. As was observed in the Canadian study, the number of chiropractors in California grew and there was drop in the doctor/patient ratio. The DCA database revealed 4274 active chiropractic licenses in 1970, which grew to 11,637 by the end of the study period in 1998. The 170% growth rate in chiropractors during the study far exceeds the 65.1% increase in residential population. As will be discussed, the disproportion between the two growth rates resulted in a decrease in the doctor/patient ratio.

**Figure 2:**
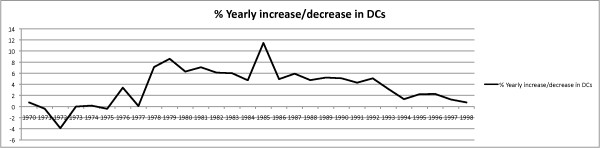
**Percentage of yearly increase/decrease in chiropractors**. The total number of licensed chiropractors decreased in 1971, 1972, 1973 and 1975, the largest drop of 3.9% occurring in 1972. The largest increase was 11.4% in 1985.

**Figure 3:**
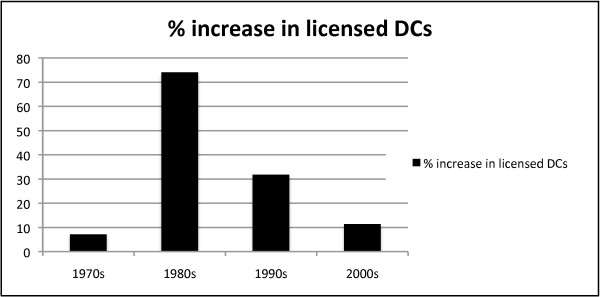
**Increase in total population of California chiropractors by decade**. A bar graph depicting the total increase in licensed chiropractors for each decade from 1970 to 2008.

The DCA database also revealed the rate of yearly percentage increase in licensed chiropractors in California between 1970 and 1998 was not linear. (Figure 2) The total number of licensees in the early portion of the 1970s actually decreased year-to-year. The total number of licensed chiropractors decreased in 1971, 1972, 1973 and 1975, the largest drop of 3.9% occurring in 1972. Large increases in licensees of 7.1% and 8.6% respectively in 1978 and 1979 eliminated the reductions seen earlier in the decade. In total, the number of licensed chiropractors rose from 4274 in 1970 to 4583 in 1979, a rise of 7.2%. Unlike the 1970s, the 1980s saw unprecedented increases in the number of licensed chiropractors. The smallest year-to-year increase of 4.7% was noted in 1984 and the largest increase was 11.4% in 1985. The DCA data revealed the number of licensed chiropractors rose from 4978 in 1980 to 8671 in 1989, a rise of 74.1% during that single decade. Although the largest surge in licensees had peaked in the 1980s, there was still an increase in licensees between 1990 and 1999. The number of licensed chiropractors rose from 9124 in 1990 to 12,043 in 1999, a rise of 31.9%. Finally, the total, the number of licensed chiropractors rose from 12,441 in 2000 to 13,822 in 2008, a rise of 11.5%. (Figure 3)

**Figure 4:**
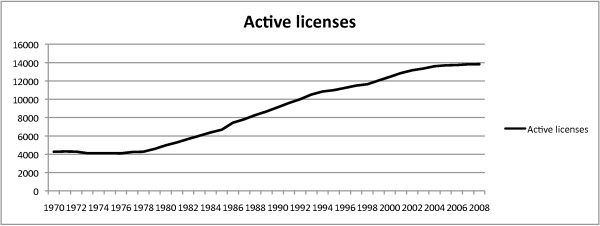
**Increase in licensed chiropractors, 1970-2008**. The number of licensed chiropractors rose from 4306 in 1970 to 11,637 in 1998, a rise of 170.2%. The total number of active licenses has continued to rise and was 13,822 in 2008.

Calculation of the growth rate in chiropractors during the 1970s, 1980s and 1990s obscures the larger impact of the total increase over the entire study period. In total, the number of licensed chiropractors rose from 4306 in 1970 to 11,637 in 1998, a rise of 170.2%. The total number of active licenses has continued to rise beyond the 1998 end of the study period and was 13,822 in 2008. (Figure 4)

**Figure 5:**
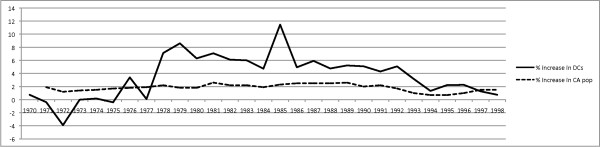
**Yearly percentage change in California population and licensed chiropractors**. The annual percentage of increase or decrease in California chiropractors from 1970 to 1998 was plotted with the solid line. The annual percentage growth in California residential population was plotted with the dashed line. The yearly percentage in licensed chiropractors exceeded the yearly percentage growth in population from 1977 until 1997. The disparity in growth rates reached its peak of 9% difference in 1985.

The overall increase in licensed chiropractors of 170.2% from 1970 to 1998 far outpaced the 65.1% growth in residential population. The percentage of change in California population on a year-to-year basis was charted and compared to the percentage of increase in the number of issued chiropractic licenses (Figure 5). Figure 5 revealed the yearly percentage in licensed chiropractors exceeded the yearly percentage growth in population from 1977 until 1997. The disparity in growth rates reached its peak of 9% difference in 1985.

The rising numbers of chiropractors, versus the available population, impacts the doctor/patient ratio. Mior and Laporte [5] documented the concurrent decrease in the doctor/patient ratio with the decrease in net annual income. They noted the ratio of population to chiropractors (total population/chiropractors = X) was 6,453:1 in 1992-93 and this ultimately dropped to 4,352:1 in 2002-2003, a reduction of 33%. Mior and Laporte determine the latter number was lower than needed for a doctor in full-time practice and contributed to their conclusion a long-term oversupply of chiropractors was present in Ontario, Canada.

We calculated the population-to-chiropractor ratio with California population numbers and the number of licensees from the DCA database. The population-to-chiropractor ratio in California was lower than that seen in Mior's Canadian study. The population of 19,971,020 and 4274 chiropractors in 1970 resulted in a population-to-chiropractor ratio of 4674:1. The population of 36,756,660 in 2008 and 13,882 chiropractors resulted in 2,647:1, a drop of 44%. These numbers are far lower than those found in Mior's study.

### Potential influences of the population-to-chiropractor ratio on attrition rates

The imbalance between the higher growth rates of chiropractors, compared to the population, is potentially significant as it dramatically changes the availability of potential patients. Previous investigations [7-10] have found a small percentage of the current population utilizes chiropractic services. Lawrence and Meeker [11] reviewed studies that analyzed chiropractic utilization and noted, "Studies looking at chiropractic utilization demonstrate that the rates vary, but generally fall into a range from around 6% to 12% of the population..." The growth in licensed chiropractors during our study resulted in a dramatic increase in competition for available patients, despite the 65% growth in residential population.

For the purposes of this study, we chose to use 10% as the percentage of the population that would utilize chiropractic care, as this falls well within the range observed in other studies cited by Lawrence and Meeker and allows for a consistent point of comparison across the study period. However, it should be noted that larger, later and more generalized studies conducted in 1999 and 2002 [12,13] concluded utilization rates of chiropractic by adults has been dropping during the later portion of the study and is now believed to be 7.5% [14].

**Figure 6:**
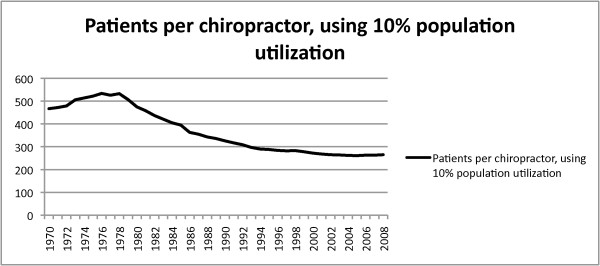
**Change in the doctor/patient ratio**. The calculated potential population for each licensed chiropractor, using 10% of the residential population, decreased from 534 patients per doctor in 1976 to 283 patients in 1998.

The results of the calculations (10% of population/licensed chiropractors = X), seen in Figure 6, reveals 467 potential patients for each chiropractor in 1970, and a peak of 534 potential patients in 1976. The potential doctor/patient ratio continued to decrease as the number of licensed chiropractors increased and outpaced population growth during the study period. The potential patient-to-doctor ratio had decreased to 283:1 by 1998, a reduction of 40% in 28 years. The rate of decline in the patient-to-doctor ratio has slowed after the study period, but currently stands at 263:1 in 2008. The slowed rate of decline in available patients is reflective of the reduced growth rate in chiropractic licenses that more closely tracks the growth in population. The authors believe the increased competition for available patients adversely affects the doctor financially, mirroring the findings in Mior and Laporte's Canadian study [5].

### Financial influences on Attrition Rates

A decrease in the population-to-chiropractor ratio appears to have a significant, and expected, negative financial effect. Mior and Laporte [5] noted the 33% reduction in the patient-to-doctor ratio in their study and observed significant decreases in the annual net income of Canadian chiropractors. Annual net income during their study dropped from $97,892 in 1992-93 to $48,900 in 2002-03, a reduction of 50% in 10 years.

The potential relationship between decreased income and increased attrition rates is obvious, but not the only area of significant financial impact. The cost of professional education and the potential for professional reimbursement are areas that have undergone significant change during our study period. Both areas were examined and indicated need for additional study.

### Tuition increases

The authors studied the change in tuition cost for the Doctor of Chiropractic degree between 1970 and 1998. Chiropractic colleges in the United States are privately funded and tuition dependent institutions that grew and changed during the study period. Expanded classrooms, improved or new facilities, increased number and quality of faculty members and costs associated with accreditation are but some of the forces that affected the cost of tuition. Increases in tuition mirrored the growth in the number of the practitioners.

Data concerning tuition for the doctor of chiropractic degree was obtained from the annual catalogues issued between 1970 and 1998 by an accredited chiropractic college in the United States. The tuition rates at this single institution were believed to be representative of tuition fees at other chiropractic colleges. An absence of older catalogs at other colleges prohibited a direct comparison. Minor fees associated with make-up tests, late registration, dissection class, graduation and transcripts were not considered as they varied between colleges and were a minor portion of the total cost.

**Figure 7:**
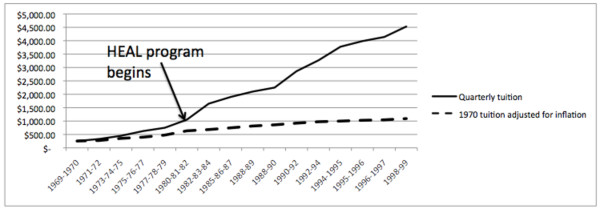
**Changes in tuition 1970 to 1999**. A time-trend depiction of the increasing quarterly tuition of a chiropractic college from 1970 to 1999. The dotted line represents the $255 per quarter tuition in 1970 adjusted for inflation between 1970 and 1999. The actual rise in tuition exceeds inflation by 414%.

Quarterly tuition in 1970, as seen in Figure 7, was $255 and rose to $4,530 in 1998, a total increase of 1,676% in 28 years. Information from the catalogs revealed the tuition increased 194% in the 1970s, 114% in the 1980s and 101% in the 1990s. The increase of tuition from the $255 per quarter level in 1970 far exceeded the rate of inflation between 1970 and 1998. The $255 quarterly tuition adjusted for inflation to 1998 dollars would have risen to $1092. Thus, the $4,530 per quarter tuition in 1998 outpaced inflation by 414%.

The rapid increase in tuition may be related to increased demand and financial rewards of practice at that time. Survey data obtained from chiropractors in the US, released by the American Chiropractic Association in 1999, [15] revealed incomes more than doubled between 1980 and 1989. Gross income rose from $83,572 in 1980 to $216,366 in 1989. Net income rose from $43,457 in 1980 to $101,423 in 1989.

The increases in earning and numbers of students enrolling in chiropractic colleges occurred simultaneously with a new source of funding for chiropractic education. In 1981, public law 97-35 made chiropractic students eligible to borrow up to $12,500 per year for four years in the Federal Health Education Loan (HEAL) program [16].

The new availability of tuition funds was mirrored by additional tuition increases and total base tuition for the DC degree in our survey college rose to $19,800 by 1982. Congress raised the total available funds through the HEAL program to $18,500 in 1992, but total base tuition for the DC degree in our survey college had now reached $39,200. By the time the HEAL program ended in 1998, the total base tuition for the DC degree had reached $54,360, a total rise of 1,676% in 28 years.

The total amount borrowed or repaid by students in California chiropractic colleges during the existence of the HEAL program is not publically available for review, but the total amount of those in default to the program is still being tracked by the US Department of Health and Human Services, Bureau of Health Professions and available for review online [17]. The debt still owed for Federal student loans to cover the cost of their education reflects a portion of the debt graduates were required to repay in a environment of increased competition. The 240 graduates of the five California chiropractic colleges who defaulted on Federal HEAL loans between 1981 and 1998 still owe a combined $20,835,958, for an average of $86,816 per person. The chiropractic profession has always represented the greatest default rate in regards to HEAL loans. It should be noted that the HEAL funds were limited to the costs for graduate education in chiropractic college and do not reflect the costs associated with undergraduate education needed for admission to chiropractic college. Those costs have also increased during the study period because of increases in pre-requisite education qualifications and rises in the costs of undergraduate courses.

### Fundamental adverse changes in healthcare reimbursement for chiropractic services occurred during the study period

Changes in the healthcare reimbursement system during the study period may have contributed to the increased attrition rate.

Unrestricted "group health" (employer provided/private payer) coverage that paid 80% of submitted chiropractic charges, and a 20% (80/20) patient co-payment was common in California in 1970 but was soon to change. Passage of the Health Maintenance Organization Act of 1973 [18] allowed for the creation of alternative reimbursement systems such as Preferred Provider Organizations (PPOs) and Health Maintenance Organizations (HMOs) that are collectively referred to as "Managed Care." The PPOs and HMOs frequently restricted access to the chiropractic profession and used fee schedules that paid a smaller percentage of the usual and customary fees, as compared to group health coverage of 80/20. Alternative reimbursement systems were authorized in 1973, but their implementation and broad adaption in society took more than a decade.

Some chiropractors elected to join networks of complementary and alternative providers that were created during the study period. Doctors joining a network were frequently required to accept the network reimbursement as payment in full. Advocates of the profession claimed these new reimbursement systems adversely affected the profession. For example, The California Chiropractic Association (CCA) filed suit against the largest network of preferred chiropractors in 2001 and alleged their practices were "damaging the entire chiropractic profession" and "negatively affected the patients' ability to access care and doctors' ability to provide care." [19] CCA further claimed the network improperly withheld 7 million dollars from network doctors between 1997 and 1999 and engaged in unfair practices. The court eventually dismissed the CCA litigation in 2005 [20]. Others in the profession documented the perceived effects of managed care on the profession. In 1994, Stephen Seater, executive director of the Foundation for Chiropractic Education and Research, stated:

"On an average day, the FCER staff talks to about 50 practicing chiropractors. These doctors are telling us more and more frequently that their practices are down, some by as much as 50%! And time and time again, the villain is managed care. In areas where managed care is growing rapidly, it is systematically cutting out most chiropractors [21]."

**Figure 8:**
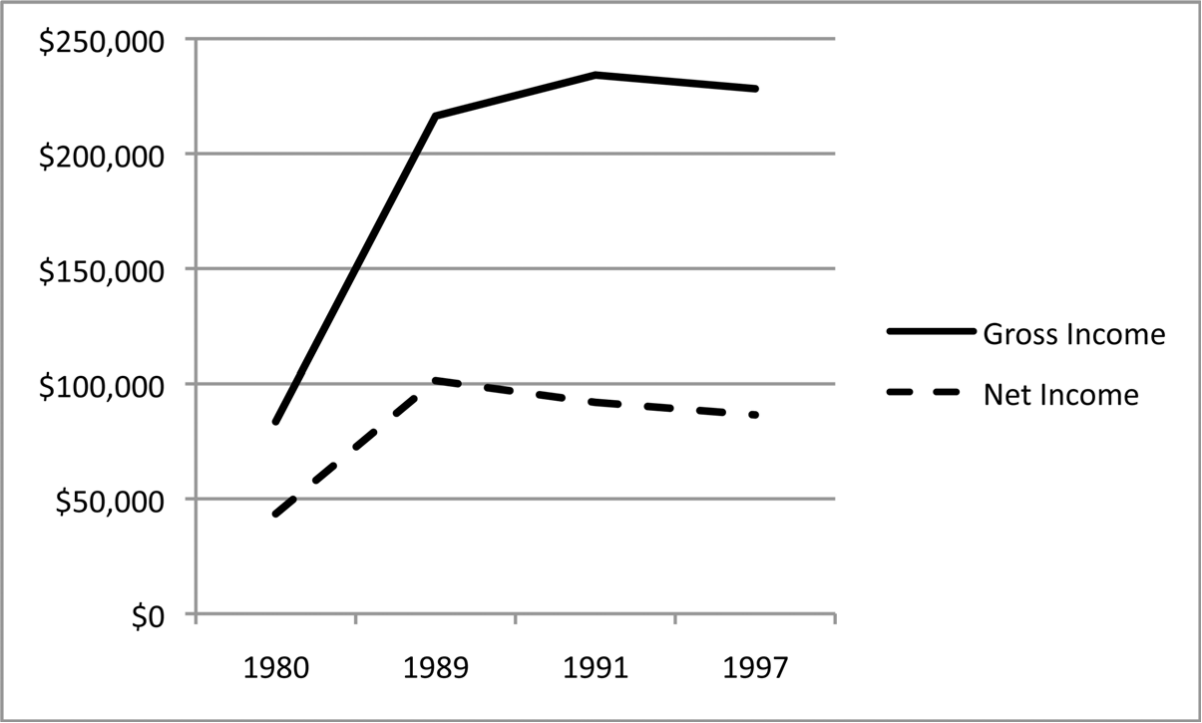
**ACA Survey data on gross and net income**. The solid line represents ACA survey data on gross income in 1980, 1989, 1991 and 1997. The dashed line represents net income from the same survey.

The claim of reduced income appears to correlate with survey data from the American Chiropractic Association [15] that showed decreases in gross and net income between 1991 and 1997 (Figure 8). The 1991 and 1997 ACA survey numbers do not account for the effects of inflation on changes of true spending power in 1997.

**Figure 9:**
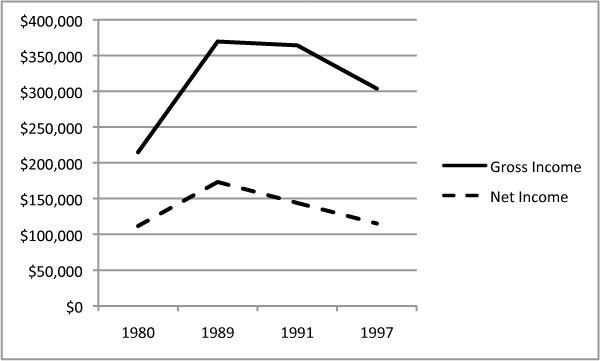
**ACA Survey data on gross and net income adjusted for inflation**. The solid line represents inflation adjusted ACA survey data on gross income in 1980, 1989, 1991 and 1997. The dashed line represents inflation adjusted net income from the same survey. Note that the adjusted net income in 1997 has returned to the 1980 level.

Figure 9 shows both gross and net incomes peaked in 1989 and decreased in spending power in both 1991 and 1997. In fact, the survey numbers show, after adjusting for inflation to 1997 levels, the net income in 1997 had returned to its 1980 level. The return to 1980 level net income was not matched by corresponding reductions in student loan debt or increases in patient-to-doctor ratios enjoyed in 1980.

## Discussion

The authors originally proposed the hypothesis that the attrition rate of licensed chiropractors is now higher than the rate seen in past decades. Analysis of the DCA records supports the hypothesis as data revealed the attrition rate to be significantly higher for those doctors licensed in the 1990s compared to their counterparts licensed in the 1970s. We also believe that the adverse financial impacts of increased competition for patients seen in other studies were mirrored in California during our study period. The 170% increase in licensed chiropractors between 1970 and 1998 far out-paced the growth in population. The exact causes of the increased rates of attrition have not been determined, but negative economic conditions produced by spiraling tuition, increased competition for available patients and lower reimbursement rates are likely contributing factors that require additional study.

There are limitations to this study. The DCA license status information does not allow determination of which doctors may have allowed their license to expire and are now practicing in other states, which doctors are in full-time versus part-time practice or the identity of doctors that may have left practice but are now employed in academia or related areas. The potential relationship between increasing attrition rates and financial factors remains hypothetical and further study would be required to establish a reliable association. Specific information concerning gross and net income levels for chiropractors within California during the study period is not available. Income observations have been limited to survey data collected from chiropractors across the United States. Tuition information has been limited to a single institution that was believed to be representative of the costs seen in other colleges. An absence of older catalogs at other colleges prohibited a direct comparison to all chiropractic colleges in California during the study. Half of the chiropractic colleges instructing students in California during the study are now closed and unable to supply information.

## Competing interests

The authors declare that they have no competing interests.

## Authors' contributions

SMF conducted the initial data extraction and prepared the first draft of the manuscript. MJS participated in the conception, review of data, and design of the study and in the revision and coordination of the final manuscript. Both authors read and approved the final manuscript.
